# The Role of Non-essential Amino Acids in T Cell Function and Anti-tumour Immunity

**DOI:** 10.1007/s00005-021-00633-6

**Published:** 2021-10-12

**Authors:** Helen Carrasco Hope, Robert J. Salmond

**Affiliations:** 1 Department of Oncology, Ludwig Institute for Cancer Research Lausanne, University of Lausanne, 1066 Epalinges, Switzerland; 2 Leeds Institute of Medical Research at St. James’s, University of Leeds, St. James University Hospital, Leeds, LS9 7TF UK

**Keywords:** Non-essential amino acid, Metabolism, T cell, Immunotherapy, Tumour microenvironment

## Abstract

T cell activation, differentiation and proliferation is dependent upon and intrinsically linked to a capacity to modulate and adapt cellular metabolism. Antigen-induced activation stimulates a transcriptional programme that results in metabolic reprogramming, enabling T cells to fuel anabolic metabolic pathways and provide the nutrients to sustain proliferation and effector responses. Amino acids are key nutrients for T cells and have essential roles as building blocks for protein synthesis as well as in numerous metabolic pathways. In this review, we discuss the roles for uptake and biosynthesis of non-essential amino acids in T cell metabolism, activation and effector function. Furthermore, we highlight the effects of amino acid metabolism and depletion by cancer cells on T cell anti-tumour function and discuss approaches to modulate and improve T cell metabolism for improved anti-tumour function in these nutrient-depleted microenvironments.

## Introduction

Naïve T lymphocytes undergo metabolic reprogramming upon antigen recognition. Specifically, activated T cells induce an anabolic-oriented metabolism that promotes the synthesis of new building blocks and allows T cells to grow, clonally expand and differentiate. Aerobic glycolysis, glutaminolysis, fatty acid synthesis and serine, glycine, one-carbon metabolism are the main metabolic pathways that are upregulated during T cell activation (Klein Geltink et al. 2018). The engagement of these pathways is regulated by the action of T cell receptor (TCR)-induced metabolic transcription factors, such as Myc (Marchingo et al. 2020; Wang et al. 2011) or hypoxia-inducible factor 1α (Phan and Goldrath 2015), and mechanistic target of rapamycin (mTOR) activity (Salmond 2018). In addition, T cell activation is accompanied by the upregulated expression of nutrient transporters that are required to fuel the aforementioned pathways. In particular, cell surface amino acid transporters are highly upregulated upon TCR-priming (Howden et al. 2019), reflecting the dependence of activated T cells on extracellular sources of these nutrients. In accordance, many studies have shown that amino acids are not only involved in protein synthesis, but also in a myriad of roles that are key to control T cell function, including epigenetic regulation, post-translational modifications, nucleotide synthesis or the maintenance of redox balance (reviewed in Kelly and Pearce 2020).

Amino acids are broadly classified as non-essential or essential depending on the body’s ability to synthesise them in sufficient quantities. In addition, several amino acids are regarded as conditionally essential and must be supplied in the diet under certain conditions such as extreme trauma or metabolic stress. Recent investigations have elucidated that de novo synthesis of non-essential amino acids is insufficient to sustain T lymphocyte function, as shown by impaired activation in the absence of extracellular supplementation. Moreover, the importance of an extracellular source of non-essential amino acids is further reinforced by the fact that T cell function is suppressed within the tumour microenvironment in part through competition with tumour cells for non-essential amino acids. In this review, we aim to summarise the role for non-essential amino acid uptake and synthesis for T cell activation and function. Furthermore, we describe the role of these amino acids in the context of anti-tumour responses and potential approaches to improve immunotherapeutic outcomes through the manipulation of non-essential amino acid metabolism in T cells and/or tumour cells.

### Glutamine

Glutamine is the most abundant amino acid in serum and the most taken up by activated T cells (Geiger et al. 2016). A key study by Nakaya and colleagues showed that CD4^+^ T cells lacking alanine serine cysteine-preferring transporter 2 (ASCT2, Slc1a5), the main glutamine transporter together with the sodium-coupled neutral amino acid transporters (SNAT) 1 and 2 (Slc38a1 and Slc38a2, respectively), displayed defective T_h_17 differentiation (Nakaya et al. 2014; Wang and Zou 2020). As a result, ASCT2-deficient mice developed a milder experimental autoimmune encephalomyelitis pathogenesis due to a reduction in numbers of interleukin-17-producing T cells (Nakaya et al. 2014). In the same study, the authors demonstrated that the lack of ASCT2 also prevented optimal T_h_1 differentiation, as shown by reduced interferon (IFN)-γ production in response to *Listeria monocytogenes* infection in vivo. ASCT2-dependent glutamine uptake was essential for TCR-induced activation of mTOR complex 1 (mTORC1) and downstream T_h_ cell differentiation.

**Fig. 1:**
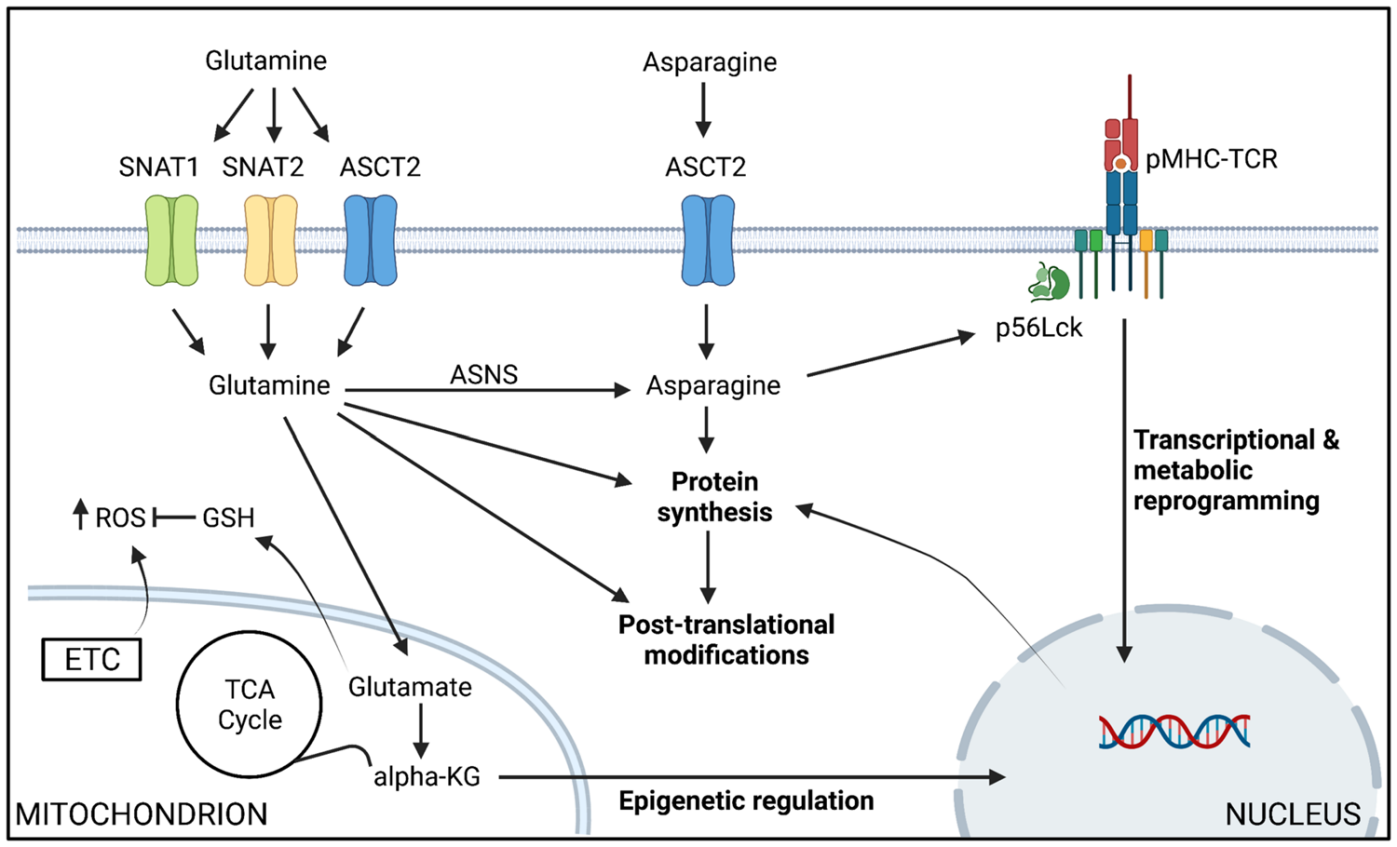
Roles of glutamine and asparagine metabolism in activated T cells. TCR-induced activation induces a transcriptional programme resulting in T cell metabolic reprogramming. Upregulation of cell surface amino acid transporters SNAT1, SNAT2 and ASCT2 facilitates elevated uptake of glutamine and asparagine from the extracellular environment. Intracellular glutamine contributes to asparagine biosynthesis via the activity of asparagine synthetase (ASNS), protein synthesis and post-translational modifications (e.g., *O*-GlcNAcylation). Glutaminolysis feeds the mitochondrial TCA cycle via the production of alpha-ketoglutarate (KG) that also contributes to epigenetic regulation during T cell activation. Furthermore, glutamine contributes to redox balance via the production of glutathione (GSH) that balances reactive oxygen species produced via the electron transport chain (ETC). Asparagine is required for protein synthesis and contributes to the regulation of TCR signalling by modulating Src-family kinase p56Lck activity. Figure created with Biorender.com

Only ~ 20% of the glutamine taken up during T cell activation is incorporated into the proteome. A major metabolic fate of glutamine within the cell is its conversion into glutamate via glutaminase activity, followed by conversion into α-ketoglutarate (Carr et al. 2010; Wang et al. 2011) (Fig. 1). This pathway, known as glutaminolysis, is key in proliferating cells to replenish the tricarboxylic acid (TCA) cycle during the engagement of aerobic glycolysis. Johnson et al. (2018) established that depleting glutaminase expression in T cells and, therefore, preventing glutamine conversion into glutamate, impeded T_h_17 cell differentiation but was beneficial for the generation of T_h_1 cells. Mechanistically, glutaminase depletion prevented the accumulation of α-ketoglutarate, that is involved in epigenetic regulation through histone methylation (Xu et al. 2017). It was shown that low α-ketoglutarate levels altered chromatin accessibility that favoured IFN-γ and T-bet gene expression. Similarly, glutaminase-depleted CD8^+^ T cells also had higher levels of T-bet and granzyme B expression, as compared to glutaminase-sufficient T cells, after activation in vitro. However, it is important to note that impairment of glutaminase activity only led to an in vivo enhancement in T cell responses when applied transiently, as T cells chronically lacking glutaminase expression became exhausted and failed to eliminate CD19-expressing B cells in a murine chimeric antigen receptor (CAR) model (Johnson et al. 2018).

The discrepancies observed in T_h_1 cell generation after depletion of ASCT2 or glutaminase activity are likely explained by the impact of ASCT2 deletion on the uptake of additional amino acids and the fact that glutamine regulates T cell activation through pathways beyond glutaminolysis. In this regard, glutamine is crucial for the synthesis of glutathione and the maintenance of redox balance (Fig. 1) (Lian et al. 2018; Mak et al. 2017). Lian et al. (2018) demonstrated that glutathione, a tripeptide formed by glutamate, cysteine and glycine, is synthesised after the activation of CD4^+^ T lymphocytes and, importantly, that this is significantly fuelled by glutamine metabolism. Disruption of glutathione synthesis through depletion of the catalytic subunit of the glutamate-cysteine ligase not only affected the activation, proliferation and viability of the cells, but also influenced their differentiation, as *Gclc*^*–/–*^ T cells favoured a regulatory T cell (T_reg_) phenotype at the expense of T_h_17 differentiation. Furthermore, a report from the Cantrell laboratory showed that a further key function for glutamine in T cells is to fuel the hexosamine biosynthetic pathway that controls the production of uridine diphosphate *N*-acetylglucosamine (UDP-GlcNAc) which is used as a substrate for protein O-GlcNAcylation (Swamy et al. 2016), a post-translational modification that occurs after T cell activation in proteins such as Myc, c-Rel and nuclear factor of activated T cells (Golks et al. 2007; Ramakrishnan et al. 2013). Additionally, glutamine participates in the synthesis of other macromolecules, such as nucleotides (Carr et al. 2010) and the non-essential amino acid asparagine (Hope et al. 2021).

These studies robustly evidence that glutamine metabolism is essential to sustain T cell function and dictate T cell fate. Indeed, effector T cells are completely unable to respond under glutamine-deprived conditions indicating that T cells cannot rely on de novo synthesis of glutamine (Carr et al. 2010; Chang et al. 2015; Klysz et al. 2015). The requirement for glutamine uptake in T cell effector responses can be observed in vivo within the tumour microenvironment as a consequence of nutrient competition. The high nutrient consumption of tumour cells generates a nutrient-deprived environment that serves as an efficient mechanism of suppressing anti-tumour responses and many current investigations are making efforts to modify the tumour microenvironment nutrient content or generating T lymphocytes that are resistant to it. Glucose and glutamine have been widely recognised as the two most important nutrients taken up by tumour cells. However, a recent report by Reinfeld et al. (2021), using positron emission tomography imaging, showed that tumour cells rely on glutamine and lipid uptake and that, unexpectedly, glucose is not a limiting factor for immune infiltrating cells. These results bring glutamine to the fore as a key metabolic target for cancer therapy.

Glutamine-restriction as an approach to cancer therapy is a challenging strategy due to its high abundance in serum. The development of glutaminase inhibitors or glutamine antagonists, such as 6-diazo-5-oxo-l-norleucine (DON), have not provided the expected outcomes during clinical trials and many have been abandoned due to unacceptable toxicities (Lemberg et al. 2018). To overcome these toxicities, Leone et al. (2019) developed the compound JHU083, an inactive form of DON that is activated by enzymes enriched within the tumour microenvironment. Tumour bearing mice treated with JHU083 presented a marked improvement of tumour growth control and survival. Most importantly, they demonstrated that targeted glutamine blockade interfered with tumour growth by acting on the tumour cells, an effect that required CD8^+^ T cell anti-tumour responses as shown by a loss of efficacy of JHU083 in *Rag2*^*–/–*^ mice or upon depletion of CD8^+^ T cells in the tumour-recipient mice (Leone et al. 2019). Other compounds such as V-9302, a selective ASCT2 inhibitor, or CB-839, a glutaminase inhibitor, have also been tested in preclinical models resulting in an amelioration of anti-tumour immunity and subsequent enhanced tumour control (Edwards et al. 2021; Reinfeld et al. 2021; Schulte et al. 2018; Varghese et al. 2021).

The utilisation of glutamine metabolism as an immunotherapeutic tool has also been explored in the context of adoptive cell therapy. Recent studies determined that glutamine restriction during CD8^+^ T cell activation in vitro promoted memory T cell differentiation, as shown by a higher development of CD44^+^CD62L^+^ cells characterised by an enhanced mitochondrial spare respiratory capacity and expression of memory-associated transcription factors, such as Tcf7 and Bcl6. The acquisition of this phenotype ultimately led to an increased number of tumour infiltrating lymphocytes upon adoptive cell transfer, added to an improved tumour growth control, survival and long-term responses (Nabe et al. 2018).

### Asparagine

The non-essential amino acid asparagine is also transported into T cells mainly via ASCT2 (Fig. 1). Asparagine can also be synthesised through the action of asparagine synthetase (ASNS), an enzyme that utilises glutamine and aspartate as substrates to generate asparagine and glutamate (Lomelino et al. 2017). Naïve T cells do not express ASNS, but it is upregulated upon TCR-stimulation in an mTOR- and Myc-dependent manner (Hope et al. 2021; Wu et al. 2021). A study performed by our group recently revealed that the dependency of T cells on an extracellular source of asparagine is inversely proportional to ASNS levels. Thus, CD8^+^ T cells primed in the absence of asparagine display defective early T cell activation associated with and likely a consequence of a reduced capacity for protein synthesis (Hope et al. 2021; Torres et al. 2016). Nevertheless, T cells lose their requirement for uptake of extracellular asparagine at later stages when ASNS is upregulated and, consequently, fully differentiated cytotoxic T lymphocytes are able to secrete cytokines even under asparagine-depleted conditions (Hope et al. 2021). Besides the requirement for asparagine in protein synthesis, another recent report demonstrated that asparagine can bind directly to the Src-family tyrosine kinase Lck, strengthening phosphorylation at its activatory Tyr394 position, thereby enhancing downstream TCR signalling and potentiating CD8^+^ T cell activity (Wu et al. 2021). Asparagine is not catabolised in mammalian cells (Pavlova et al. 2018; Wu et al. 2021) but whether asparagine could interact with other proteins and influence T cell function through to date unknown mechanisms needs to be elucidated.

In the context of cancer metabolism, asparagine has been described as an important ally of glutamine. Pavlova and colleagues showed that asparagine can rescue tumour cell survival in settings where glutamine availability is compromised (Jiang et al. 2018; Pavlova et al. 2018). Similarly, the availability of extracellular asparagine can partially compensate for glutamine-deprivation in maintaining T cell survival whilst combined asparagine and glutamine deprivation results in very high levels of T cell death (Hope et al. 2021). In tumour cells cultured in glutamine-limited conditions, asparagine promotes protein synthesis and induces the expression of glutamine synthetase, restoring mTOR activity and cell proliferation (Pavlova et al. 2018). Furthermore, asparagine also acts as an exchange factor for the uptake of other amino acids, such as leucine, serine or threonine, which subsequently boosts mTORC1 activation, the synthesis of nucleotides and amino acids and cancer cell proliferation (Krall et al. 2016). Additionally, Knott et al (2018) showed that expression of ASNS is a determinant of metastatic potential in the 4T1 mouse model of breast cancer. Moreover, they showed that the supplementation of exogenous asparagine, but not other non-essential amino acids, promoted the invasiveness of 4T1 cells (Knott et al. 2018). According to these reports, exploiting asparagine bioavailability might limit tumour growth and metastasis in vivo. Indeed, the use of asparaginases has been used in the clinic for the treatment of acute lymphoblastic leukaemia that express low levels of ASNS and are highly rely on extracellular asparagine (Chiu et al. 2019). However, the efficacy of asparaginases decreases when applied to other solid tumours, as these can compensate the loss of exogenous asparagine expressing high levels of ASNS (Chiu et al. 2019). This suggests that both exogenous and ASNS-derived sources of asparagine should be targeted to efficiently dampen asparagine dependence of tumour cells. Krall et al. (2021) followed this rationale and showed that tumour growth was more efficiently controlled when treating tumour-bearing mice with asparaginases in combination with metformin, an inhibitor of the electron transport chain that diminishes the levels of the ASNS-substrate aspartate. Whether this therapeutic combination might negatively affect T cell-mediated anti-tumour immunity is less understood. Wu et al. (2021) provided evidence that, in some circumstances, dietary asparagine depletion is favourable for tumour growth, as the initiation of CD8^+^ T cell responses in vivo are impeded in the absence of asparagine. Further study is required to fully comprehend the impact of asparagine bioavailability in both tumour cells and anti-tumour immunity and to determine an appropriate approach to exploit asparagine metabolism in cancer therapy.

### Arginine

Arginine can be synthesised from ornithine through arginosuccinate synthase (ASS1). However, T cells do not express ASS1 and are highly dependent on arginine uptake mainly via the high affinity cationic amino acid transporter (CAT)-1 (Slc7a1) (Crump et al. 2021; Wang and Zou 2020). Thus, T cells activated in vitro in arginine-free media are unable to undergo antigen-induced blastogenesis or proliferation and display defective CD4^+^ T cell differentiation and cytokine secretion even under polarising conditions (Crump et al. 2021; Wu et al. 2020a).

**Fig. 2:**
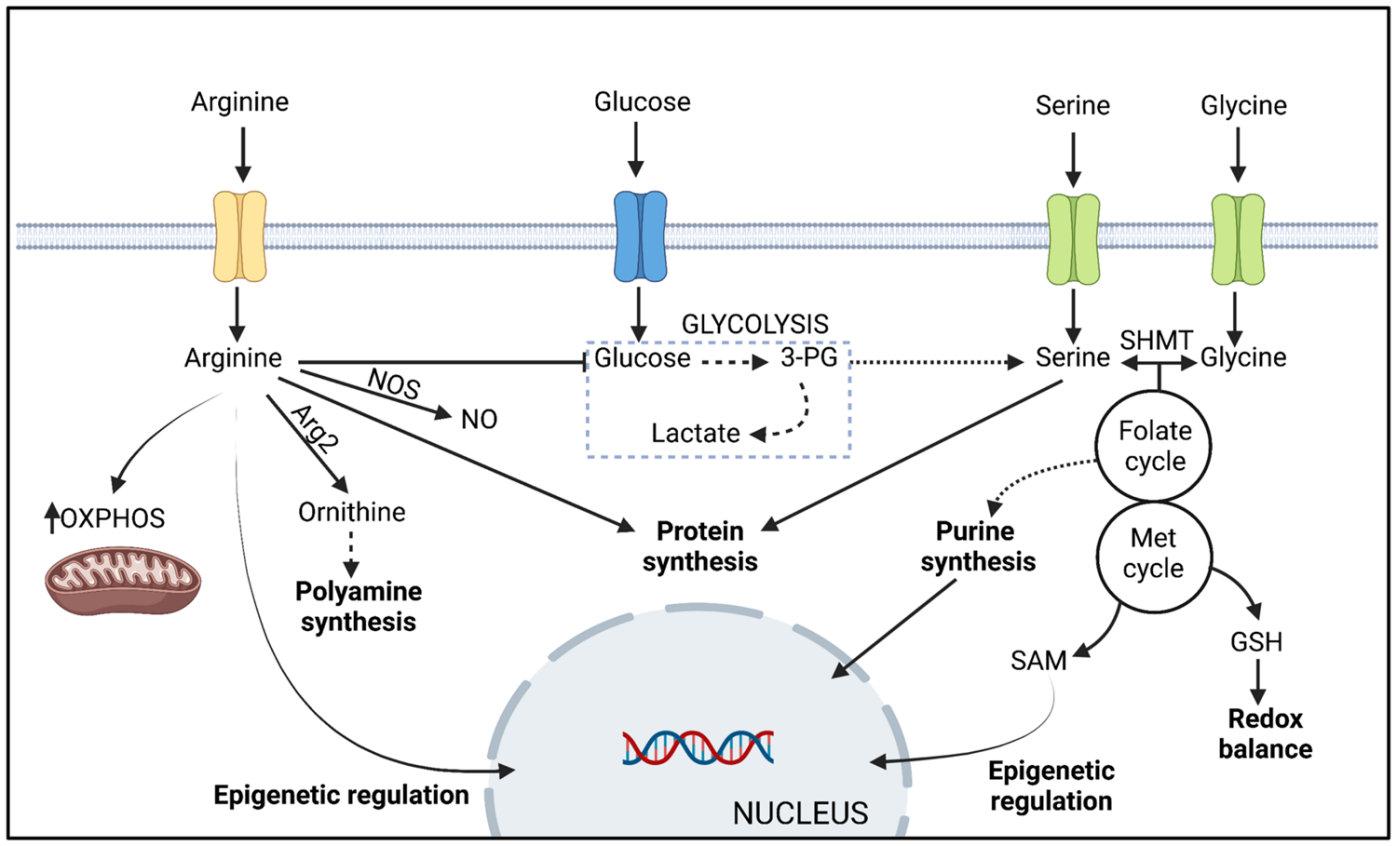
Metabolic functions of arginine, serine and glycine in T cells. Arginine, serine and glycine all serve as building blocks for protein synthesis. Arginine serves as the major carbon donor for polyamine synthesis and is required for nitric oxide (NO) production via the activities of arginase 2 (Arg2) and nitric oxide synthase (NOS), respectively. High levels of intracellular arginine skew cellular metabolism, enhancing mitochondrial oxidative phosphorylation (OXPHOS) whilst dampening glycolysis. Arginine contributes to epigenetic regulation in tumour cells, although this function has yet to be fully defined in T cells. The glycolytic metabolite 3-phosphoglycerate (3-PG) serves as a building block for serine biosynthesis, whilst serine hydroxymethyltransferases (SHMT) catalyse the interconversion of serine and glycine. Serine and glycine one-carbon metabolism contributes to purine nucleotide biosynthesis via the folate cycle and the production of glutathione (GSH) via the methionine (Met) cycle. S-adenosyl methionine (SAM), derived from the methionine cycle, is a methyl donor for methyltransferases involved in epigenetic regulation. Figure created with Biorender.com

Arginine is the major carbon donor for polyamine synthesis (Wu et al. 2020a). Interestingly, arginine is the only amino acid of which the intracellular concentration drops after T cell activation (Geiger et al. 2016). The reason why this occurs is not due to a limited uptake via CAT-1, but rather due to a rapid metabolism of arginine into ornithine through the action of arginase 2 (Arg2) which, in turn, serves as a substrate for the synthesis of proline, glutamate and polyamines such as putrescine or spermidine (Geiger et al 2016; Wu et al. 2020a). Nevertheless, Wu et al. (2020a) demonstrated that de novo polyamine synthesis is dispensable for T cell responses in vivo*,* as shown by unchanged experimental autoimmune encephalomyelitis pathogenesis in mice with T cell-specific deletion of ornithine decarboxylase. This data suggest that arginine might be implicated in other T cell functions. Indeed, beyond contributing to protein synthesis, the Lanzavecchia laboratory reported that arginine can interact with the transcriptional regulators BAZ1B, PSIP1 and TSN leading to an improved mitochondrial fitness and T cell survival (Geiger et al. 2016). Thus, elevated intracellular levels of arginine favoured mitochondrial oxidative phosphorylation in activated human T cells and thereby skewed metabolism away from glycolysis. More recently, an investigation by Crump et al. (2021) described that T lymphocytes primed in the absence of extracellular arginine are incapable of promoting chromatin remodelling induced upon T cell activation, suggesting a role for arginine as a regulator of epigenetic modifications. Arginine also functions as a substrate to produce nitric oxide via nitric oxide synthases (NOS). Indeed, recent evidence has suggested that cell-intrinsic expression of NOS2 is required for the stability of human T_h_17 cells (Obermajer et al. 2013). The major roles for arginine in T cell metabolism are summarised in Fig. 2.

With regards to the roles for arginine metabolism during anti-tumour responses, tumour-associated cells (e.g., fibroblasts, tumour-associated macrophages and myeloid-derived suppressor cells) or cancer cells themselves, increase arginase expression as a mechanism of immunological tolerance (Timosenko et al. 2017). Whilst tumour cells can metabolically adapt to arginine deprivation by upregulating ASS1 in an ATF4 and CEBPβ-dependent manner, T lymphocytes become dysfunctional in this context (Crump et al. 2021). By contrast, genetic ablation of *Arg2* expression in mice increases arginine levels in serum, secondary lymphoid organs and within the tumour microenvironment leading to an enhanced tumour infiltration of CD8^+^ T cells that results in a slower growth of MC38 colon carcinoma or B16 melanoma (Marti i Lindez et al. 2019). In the same study, the authors showed T cell-specific deletion of *Arg2* resulted in enhanced anti-tumour responses upon adoptive cell transfer, as compared to wild-type T cell adoptive transfer, an effect that was even more profound when combined with immune checkpoint blockade. Another report demonstrated that T cell expansion in arginine-rich conditions promoted the engagement of mitochondrial metabolism at the expenses of glycolysis, generating “memory-like” T lymphocytes and providing prolonged anti-tumour responses after adoptive cell transfer (Geiger et al. 2016). More recently, Fultang et al. (2020) generated ASS1-expressing CAR-T cells that showed enhanced T cell proliferation in vitro in addition to improved tumour control and in vivo survival. These investigations provide strong evidence that arginine metabolism plays a key role in the outcome of anti-tumour immunity, offering a promising target for immunotherapy.

### Serine and Glycine

Serine is synthesised intracellularly via a pathway initiated by the oxidation of the glycolytic intermediate 3-phosphoglycerate by the enzyme phosphoglycerate dehydrogenase (PHGDH) (Fig. 2). In turn, the conversion of serine to glycine is catalysed by serine hydroxymethyltransferases (SHMT). Ma et al. (2019), using stable isotope tracing, demonstrated an elevated flow of glucose carbon to serine biosynthesis in effector T cells in vivo. Furthermore, PHGDH expression was important for T cell proliferation both in vitro and in vivo. Despite this requirement for glucose-derived serine in T cell activation, evidence indicates that T cell responses also require extracellular sources of this non-essential amino acid. In this regard, dietary restriction of serine limited T cell responses in a model of *L. monocytogenes* infection (Ma et al. 2017) whilst serine restriction combined with PHGDH knockdown completely prevented T cell proliferation in vitro (Ma et al. 2019).

The serine, glycine, one-carbon metabolic network enables cells to convert serine and glycine to a number of metabolites that sustain biosynthesis pathways and contribute to redox balance and epigenetic regulation (reviewed in Reina-Campos et al. 2020; Wu et al. 2020b). The Jones laboratory determined that upregulated expression of serine, glycine, one-carbon pathway enzymes was a feature of activated T cells (Ma et al. 2017). Pharmacological inhibition of both mitochondrial and cytosolic SHMT isoforms, thereby blocking serine to glycine conversion, had a substantial impact on purine nucleotide synthesis and impeded T cell proliferation, without impacting cell viability. Therefore, a key function of serine, glycine, one-carbon metabolism in T cells is in maintaining proliferative responses through contributions to nucleotide synthesis (Fig. 2).

Increasing evidence points towards a key role for serine bioavailability and de novo serine biosynthesis in the regulation of tumour growth in vivo. Dietary restriction of serine and glycine reduced tumour growth and increased survival in autochthonous mouse models of lymphoma and intestinal adenoma (Maddocks et al. 2017). Nonetheless, not all mouse tumour models were sensitive to serine-glycine restriction, presumably due to an increased capacity for serine biosynthesis. Consistent with this, increased *PHGDH* copy number is a feature of triple-negative breast cancers and melanomas, whilst brain metastases demonstrate particularly high levels of expression of PHGDH and other SGOC enzymes (Ngo et al. 2020). It is likely that an elevated capacity for glucose-dependent serine biosynthesis confers a growth advantage in tissues in which serine levels are limiting (Maddocks et al. 2017; Sullivan et al. 2019). Indeed, targeting PHGDH using small molecule inhibitors limited growth of breast cancer brain metastases in mouse models (Ngo et al. 2020). As yet, the impact of such interventions on anti-cancer immune responses remains to be determined. Furthermore, the impact of cancer cell consumption of serine and/or glycine on T cell function within the tumour microenvironment has yet to be directly assessed.

### Other Non-essential Amino Acids

The roles for asparagine, arginine, serine, glycine and, in particular, glutamine in T lymphocytes and anti-tumour responses are well studied. The metabolism and uptake of other non-essential amino acids are also likely to play an essential role in immunity. For example, Ron-Harel et al. (2019) recently reported that uptake of alanine is required for CD8^+^ T cells to exit quiescence, both in naïve and memory states. In this study, the authors demonstrated that only 1–2% of the alanine pool in early activated T cells is synthesised de novo from pyruvate via glutamate-pyruvate transaminase. Moreover, they showed that alanine was not further catabolised and that it mainly contributed to T cell function through the synthesis of new proteins (Ron-Harel et al. 2019).

Another important non-essential amino acid in T cells is cysteine. Cysteine can be derived from methionine but is primarily taken up by the cystine-glutamate antiporter (xCT, Slc7a11), that has been shown to be upregulated upon activation of human CD4^+^ and CD8^+^ T cells (Siska et al. 2016). A study by Srivastava et al. (2010) demonstrated that one of the mechanisms by which myeloid-derived suppressor cells inhibit T cell activation is by sequestration and subsequent depletion of cysteine, highlighting the importance of an extracellular source of this non-essential amino acid. Interestingly, decreasing the cysteine concentration in in vitro cultures limited CD4^+^ T cell polarisation to a T_h_17 phenotype, with no impact on T_h_1, T_h_2, or iT_reg_ differentiation, indicating a selective role for cysteine uptake in CD4^+^ T cell responses (Sundrud et al. 2009). Besides protein synthesis, the major roles of cysteine in T cells are in regulation of redox balance through the synthesis of glutathione but also its capacity to act as a sulphur donor for example in iron-sulphur clusters, which are important components of enzymes that form the electron transport chain, thereby indirectly affecting mitochondrial metabolism. Recent studies have shown that targeting cysteine-cystine uptake may represent a rational target for the treatment of pancreatic cancers (Badgley et al. 2020). Depletion of Slc7a11 in pancreatic duct adenocarcinoma cells or depletion of cysteine/cystine using cysteinases resulted in cell death by ferroptosis, as a consequence of depletion of glutathione (Badgley et al. 2020). These results suggest that cancer cells require extracellular cysteine within the tumour microenvironment, although further studies are required to confirm the importance of tumour cell-mediated depletion of this amino acid on infiltrating T cell function.

The small molecule halofuginone, a synthetic quinazolinone, limits T_h_17 responses by inducing the amino acid response (Sundrud et al. 2009) whilst anti-proliferative effects of the compound were linked to decreased T cell uptake of the non-essential amino acid proline (Chu et al. 2013). Although relatively uncharacterized in T cells, proline metabolism has a vital and complex role in tumour progression and metastasis (Burke et al. 2020). For example, Elia et al. (2017) reported that CRISPR-Cas9 deletion of proline dehydrogenase (PRODH) and inhibition of proline catabolism reduced ATP production in breast cancer cell spheroids and impaired lung metastasis formation in mouse models. Although the tumour-promoting or suppressing role of PRODH in tumour cells is highly context dependent, recent studies showed that prostate cancer cells suppressed T cell responses via the metabolite 1-pyrolline-5-carboxylate, a downstream product of proline catabolism by PRODH (Yan et al. 2018).

Recent studies have highlighted the importance of aspartate biosynthesis in proliferating cells (Birsoy et al. 2015; Sullivan et al. 2015) as well as in T cell activation and differentiation (Bailis et al. 2019). Bailis et al. (2019) demonstrated that the malate-aspartate shuttle was required to support complex I of the electron transport chain and subsequent T cell activation. Inhibition of the electron transport chain with rotenone inhibited aspartate biosynthesis and induced a G2/M cell cycle block, that was partially relieved by supplementation of T cells with extracellular aspartate (Bailis et al. 2019).

Tyrosine is synthesized intracellularly from the essential amino acid phenylalanine via the activity of phenylalanine hydroxylase. Tyrosine is a proteinogenic amino acid whilst phosphorylation of tyrosine residues by protein tyrosine kinases play a key role in signal transduction and T cell activation (Salmond et al. 2009). Suppression of tumour-infiltrating lymphocyte function in prostate cancer was associated with increased nitration of protein tyrosine residues and reversed by the use of arginase or NOS inhibitors (Bronte et al. 2005). Although the role of some of these non-essential amino acids and their downstream metabolism has been explored in cancer cells, the crosstalk with immune cells remains poorly understood. Further investigation is needed to fully understand how the availability and metabolism of these non-essential amino acids regulate anti-tumour responses.

## Concluding Remarks

In T lymphocytes, amino acids are involved in multiple functions that include, among others, the provision of building blocks for proteins, nucleotides and lipids, the regulation of the epigenome and the maintenance of redox balance that are key to sustain T cell function and finely tune T cell differentiation. As a consequence, amino acids are highly demanded by activated T lymphocytes converting non-essential amino acids into conditionally essential nutrients. Similarly, cancer cells also strongly rely on non-essential amino acid uptake to support growth and dissemination, leading to nutrient competition within the tumour microenvironment that deprives immune infiltrating cells of non-essential amino acids and contributes to T cell dysfunction. Approaches that reinvigorate the uptake and metabolism of non-essential amino acids in T lymphocytes, either by dietary modifications or the ex vivo generation of T cells resistant to the nutrient restricted tumour microenvironment, have been shown to successfully ameliorate anti-tumour T cell responses and control tumour growth in preclinical studies. Further studying the role of non-essential amino acid metabolism in both T lymphocytes and cancer cells will greatly enhance our understanding of the immunometabolic mechanisms that regulate anti-tumour immunity and will shed light into novel targets for cancer therapy.
